# Discovery of the order ‘*Quisvirales*’ redefines the evolution of RNA replication and transcription in the phylum *Pisuviricota*

**DOI:** 10.1093/ve/veag052

**Published:** 2026-08-18

**Authors:** Alexander E Gorbalenya, Dmitry V Samborskiy, Benjamin W Neuman, Chris Lauber

**Affiliations:** Leiden University Center of Infectious Diseases, Leiden University Medical Center, Albinusdreef 2, 2333 ZA Leiden, the Netherlands; Belozersky Institute of Physico-Chemical Biology, Lomonosov Moscow State University, Leninskie Gory 1, 119992 Moscow, Russia; Department of Biology, Microbial Pathogenesis & Immunity, Texas A&M University, 525 Lubbock St, College Station, TX 77843, United States; Institute for Experimental Virology, TWINCORE Centre for Experimental and Clinical Infection Research, a joint venture between the Hannover Medical School (MHH) and the Helmholtz Centre for Infection Research (HZI), Feodor-Lynen-Str. 7, 30625 Feodor-Lynen-Str. 7, 30625 Hannover, Germany; Cluster of Excellence 2155 RESIST, Hannover Medical School (MHH), Carl-Neuberg-Str. 1, 30625 Hannover, Germany

**Keywords:** *Quisvirales*, RNA virus replicase, helicase superfamily, AAA+/RecA-like ATPase, nidovirus evolution, discontinuous transcription

## Abstract

Genome replication in positive-stranded RNA (ssRNA+) viruses is mediated by cognate enzymes, including ubiquitous RNA-dependent RNA polymerase (RdRp). In ssRNA+ viruses with multiple open reading frames (ORFs) in their genomes, replication often is accompanied by synthesis of subgenomic RNAs (transcription) for expression of 3′-proximal ORFs. In addition, all ssRNA+ viruses with genomes larger than ~7 kb encode helicases, linking helicases to RNA genome expansion. Helicases are essential ATPases that unwind nucleic acids and are classified into six recognized superfamilies (SF1–SF6). In the phylum *Pisuviricota* that includes important pathogens, helicases of SF1–SF3 are integrated into multi-enzyme replicase polyprotein(s) including 3C(-like) protease (3CLpro) and RdRp. Here, large-scale mining of invertebrate metatranscriptomes and targeted genome sequence assembly uncovered six spider-associated ssRNA+ viruses that, based on their conserved 3CLpro–RdRp module in replicase polyproteins, genome size (20–22 kb), and phylogeny, form a family-like cluster in a putative order, named ‘*Quisvirales*’. Quisviruses have similar genome and replicase architectures to enveloped coronaviruses and other nidoviruses. Notably, quisviruses encode ORFs 1a and 1b with predicted -1 programmed ribosomal frameshifting elements in the ORF1a/b overlap region. Using an original mapping approach for detecting chimeric sequencing reads, we obtained evidence that 3′-proximal ORFs are expressed *via* 5′-coterminal, leader-containing subgenomic RNAs. This suggests that the quisvirus subgenomic RNAs are generated through discontinuous transcription—a mechanism otherwise exclusively found in nidoviruses among the many ssRNA+ virus orders that synthesize subgenomic RNAs. Striking differences between nido- and quisviruses are, however, the RdRp being the only common core ORF1b-encoded enzyme and the replacement of the nidovirus SF1 helicase by a novel superfamily helicase. This quisvirus SF7 helicase, like the *Picornavirales* SF3 helicase, comprises an AAA+ (ATPase-like) domain typical for ring-forming helicases and thus must play an essential role in replication. The discovery of the order ‘*Quisvirales’* demonstrates that viruses employing large replicase polyproteins of nidovirus-like complexity and discontinuous transcription may have evolved repeatedly from an 3CLpro–RdRp-encoding ancestor.

## Graphical Abstract

**Graphical Abstract:**
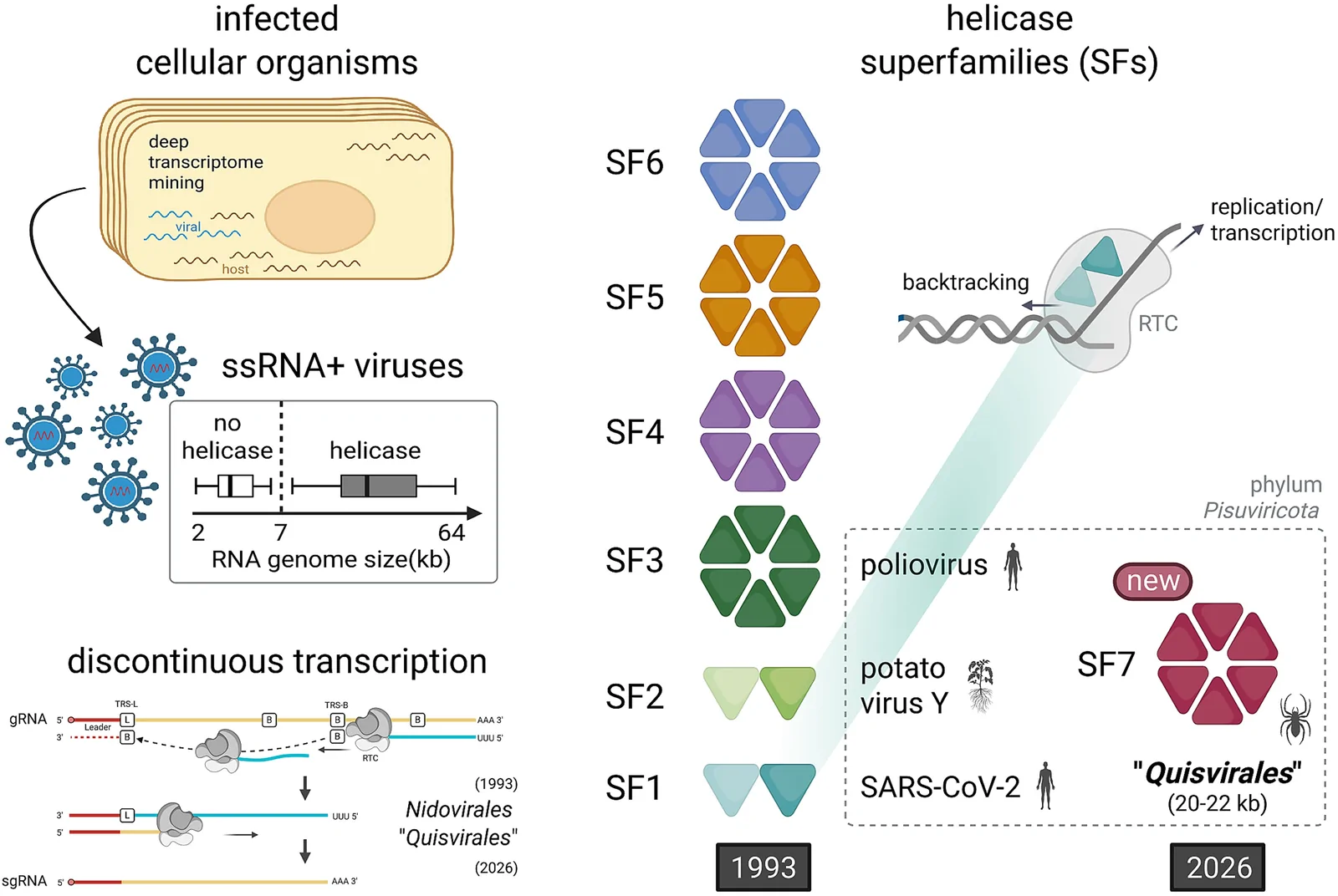

## Introduction

All RNA viruses replicate their genomes using cognate enzymes that may also mediate synthesis of subgenomic RNAs (transcription) in multi-cistronic viruses (Ball 2001). The RNA-dependent RNA polymerase (RdRp) is the hallmark protein of RNA viruses with no DNA stage during replication and the principal nucleic acid–processing enzyme required for viral genome replication and transcription (Mutz et al. 2025). It may be assisted by other virus enzymes and cofactors as part of the viral replication–transcription complex (RTC), with the RTC complexity increasing with RNA genome size (Den Boon et al. 2010, Lauber and Gorbalenya 2016, Malone et al. 2022, Madhugiri et al. 2026). In all known positive-stranded RNA (ssRNA+) viruses with documented proteome and a genome size of >7.9 kb and some with smaller genomes starting with 5.4 kb, the RdRp is supported by a cognate essential helicase in the membrane-bound RTC (Gorbalenya and Koonin 1989, Úbeda et al. 2025). Helicases may be indispensable for replication and expression of (relatively) large RNA genomes and are among the most common enzymes of RNA viruses (Den Boon et al. 2010, Grimes and Denison 2024, Mutz et al. 2025, Madhugiri et al. 2026). They are ATP-hydrolysing motor proteins (ATPases) that move directionally along single- or double-stranded DNA or RNA molecules while displacing a single-stranded polynucleotide or subunit of protein complexes (Lohman and Bjornson 1996, Pyle 2008). Universal features of all known helicases include three conserved sequence motifs, two involved in binding and hydrolysis of NTP (Walker A and B boxes) and one containing an arginine finger for energy coupling to the helicase displacing activity (Singleton et al. 2007). Helicases may act on DNA, RNA, or DNA–RNA hybrid templates; move from 5′- to 3′-end or reverse direction; and have been associated with a vast variety of functions in (infected) cells.

Based on sequence and structural similarities and mechanistic differences, helicases have been classified into six superfamilies (SF1 to SF6) present in DNA-based life forms, three of which (SF1, SF2, and SF3) are also found in ssRNA+ viruses (Gorbalenya and Koonin 1993, Singleton et al. 2007). The core of SF1 and SF2 helicases is composed of two paralogous subdomains with RecA-like folds encompassing seven to eight sequence motifs and the N-terminal subdomain being a P-loop ATPase (Iyer et al. 2004). The active form of SF1/SF2 helicases is the monomer, although they may also form dimers (Nanduri et al. 2002, Brendza et al. 2005, Dillingham et al. 2005, Zhang et al. 2006). Translocation of SF1/SF2 helicases along the DNA/RNA template is assumed to follow an inchworm-like mechanism (Yarranton and Gefter 1979). In contrast, the core of SF3–6 helicases is twice smaller and comprises a single P-loop NTPase domain, either of RecA-like fold (SF4–5) or of related AAA+ domain (ATPases associated with diverse cellular activities) in SF3 and SF6 (Iyer et al. 2004, Hickman and Dyda 2005, Itsathitphaisarn et al. 2012). It includes four to six sequence motifs, four of which are the universal Walker A and B boxes, a sensor-1 and an Arg finger, with SF-specific spacing. Helicase molecules of SF3–6 assemble into a homo-oligomeric rings that may undergo oligomeric size transition and are the active enzymatic form (Patel and Picha 2000, Enemark and Joshua-Tor 2006, Singleton et al. 2007). Translocation of SF3–6 helicases is proposed to follow a hand-over-hand mechanism in which sequential ATP hydrolysis by subunits causes ring rotation coupled with its movement along the template (Itsathitphaisarn et al. 2012).

Helicase-encoding RNA viruses belong to several orders and three families of the phyla *Kitrinoviricota* and *Pisuviricota* (kingdom *Orthornavirae*) (Koonin et al. 2020*)*. In *Pisuviricota*, the proteome of orders *Nidovirales* and *Picornavirales* (class *Pisoniviricetes*) and *Patatavirales* (class *Stelpaviricetes*) include helicases of SF1, SF3, and SF2, respectively, either upstream or downstream of the essential 3C(-like) protease (3CLpro) and protein-primer-dependent RdRp in replicase polyprotein(s). These viral enzymes were predominantly delineated by comparative genomics, and the observed patterns are order-specific. 3CLpro comprises a subset of proteases with chymotrypsin-like fold, Ser/Cys as the catalytic nucleophile, and active site features that define a conserved narrow substrate specificity. It controls genome expression by mediating autoproteolytic processing of polyprotein(s) into subunits of the replication–transcription complex; in viruses that use jelly-roll capsids, it also cleaves precursors of capsid subunits (Gorbalenya and Snijder 1996, Flint and Ryan 1997, Tong 2002). There are two other 3CLpro-RdRp-encoding ssRNA+ orders, *Sobelivirales* (class *Pisoniviricetes*) and *Stellavirales* (class *Stelpaviricetes*), that include viruses with genome sizes under 7.9 kb and no helicase encoded (Bosch et al. 2012, Sõmera et al. 2021, Gracy et al. 2024, Noyvert et al. 2026).

Viruses of the order *Nidovirales* have evolved RNA genomes in the range from 12 to 64 kb and employ complex replication and expression strategies in vertebrate and invertebrate hosts (Malone et al. 2022, Neuman et al. 2025, Zhong et al. 2025, Madhugiri et al. 2026). Members of three most characterized nidovirus families, *Arteriviridae, Coronaviridae,* and *Tobaniviridae*, produce 5′- and 3′- coterminal, leader-containing subgenomic (sg) RNAs. They use a unique mechanism of discontinuous transcription of the 3′-terminal genomic regions encoding open reading frames (ORFs) for virion and accessory proteins, which has prompted the name *nido*virus (nested) for the order (Cavanagh 1997, Van Vliet et al. 2002, Pasternak et al. 2006, Sawicki et al. 2007, Sola et al. 2015). This mechanism is guided by conserved leader and body transcription regulatory sequences (TRSs) in the vicinity of the respective initiator AUG codon (Di et al. 2017, Sola and Zuñiga 2026), although two overlapping ORFs may be expressed from an sg mRNA under translational control (Firth and Brierley 2012). While discontinuous transcription is restricted to nidoviruses, continuous transcription functions in multi-cistron viruses of *Sobelivirales, Stellavirales, Picornavirales,* and several other orders of the phylum *Kitrinoviricota* (Miller and Koev 2000, Sztuba-Solińska et al. 2011). This considerable disparity in utilization suggests that discontinuous transcription might have emerged only once and may be associated with specifics of nidovirus replication and transcription.

Described some 35 years ago, helicase classification and domain linkages still hold in many thousands of the newly discovered viruses from an ever-expanding host range of uni- and multi-cellular eukaryotes (Culley et al. 2006, Lauber and Gorbalenya 2016, Shi et al. 2016a, Hou et al. 2024, Mutz et al. 2025). They imply that the observed genomic and phylogenetic patterns must have emerged early in the evolution of ssRNA+ viruses. Also, they reveal evolutionary constraints on 3CLpro-RdRp-encoding viruses, especially in relation to helicases. The 3CLpro-RdRp-encoding viruses represent an evolutionarily successful subset of RNA viruses, since they cover a nearly full range of the documented RNA genome sizes from 4 to 64 kb and have explored diverse genomic organizations and expression mechanisms during evolution (Zell et al. 2017, Olendraite et al. 2019, Vinjé et al. 2019, Brinton et al. 2021, Lang et al. 2021, Sõmera et al. 2021, Fuchs et al. 2022, Inoue-Nagata et al. 2022, Neuman et al. 2025). The phylum *Pisuviricota* includes important pathogens of humans, e.g*.* SARS-CoV-2, poliovirus, and norovirus.

Here, we delineate a putative ssRNA+ virus order ‘*Quisvirales*’ in the class *Pisoniviricetes,* based on RdRp phylogeny and presence of the 3CLpro-RdRp module in genome sequences assembled from spider metatranscriptomes. Quisviruses have large, multi-ORF-encoding RNA genomes in the size range of 20–22 kb and putative multi-enzyme replicase polyproteins of large nidovirus-like complexity. Strikingly, the nidovirus parallel extends to the 3′-terminal ORFs, which may be expressed through discontinuous transcription. However, and contrary to the observed domain linkages in relation to genome size, quisviruses do not encode a helicase of known superfamilies. Instead, we identified a novel AAA+ domain that is conserved in six known quisviruses in the genomic position of nidovirus SF1 helicase. Like ring-forming helicases of SF3–6, this putative ATPase is predicted to adopt a single RecA-like fold at its core and includes four key sequence motifs. Its evolutionary distances from SF1 to 6 helicases are as large as between different SFs. We propose that this ATPase is the prototype of a new helicase superfamily and may play a key role during replication of quisviruses.

## Methods

### Virus discovery from public sequence databases

We applied a data-driven virus discovery approach using the Virushunter and Virusgatherer workflows to screen 30 105 arthropod transcriptome sequencing projects from the Sequence Read Archive (SRA) for the presence of RNA virus genomes, as described previously (Lauber et al. 2017, Chong and Lauber 2023, Lauber et al. 2024, Neuman et al. 2025). Among the hits were two divergent viral genomes that clustered together and otherwise had highest sequence similarity to members of the order *Picornavirales* and other pisoniviruses in a protein BLAST search (blastx) against the National Center for Biotechnology Information (NCBI) viral_protein database. They constitute the first two representatives of quisviruses, recognized and named in this study (see below). We used the predicted RdRp-encoding polyproteins of these two viral genomes as query in a protein-level Blast search (tblastn with default parameters), conducted at the NCBI website, against all Arthropoda sequences from the Transcriptome Shotgun Assembly (TSA) database (Clark et al. 2016). We considered hits with an E-value of 1 × 10^−50^ or better and a contig length of at least 10 kb. This TSA screening approach yielded four additional quisvirus hits. These four viral genomes were re-assembled from the corresponding raw-read SRA data using Megahit with default parameters (Li et al. 2015). We obtained several additional strongly supported hits against contigs shorter than 10 kb in different spider projects, which were not considered for further analysis in this study. Details about the six viral genomes and the datasets in which they were discovered can be found in Supplementary Table S1.

### Viral genome organization

ORFs were predicted using getorf from the EMBOSS package (Rice et al. 2000), considering ORFs from start to stop codons of at least 300 nucleotides, except for ORF1b that was delimited from stop to stop codons. Programmed ribosomal frameshifting elements were predicted using KnotInFrame (Theis et al. 2008).

### Protein domain annotation

Quisvirus protein domain borders were tentatively delineated using multiple sequence alignment to reference RNA genomes and their annotated proteins in the Viralis software (Gorbalenya et al. 2010). Functional annotation of quisvirus proteins involved profile Hidden Markov Model (HMM) comparisons to viral and host reference proteins from the Protein Data Bank (PDB), Pfam-A, Uniprot, and NCBI Conserved Domains databases utilizing HHpred searches at the MPI Bioinformatics Toolkit web service (https://toolkit.tuebingen.mpg.de) (Alva et al. 2016, Marchler-Bauer et al. 2017, wwPDB consortium et al. 2019, Mistry et al. 2021, The UniProt Consortium et al. 2023) or as described in our research (Neuman et al. 2025).

### Phylogenetic analysis

We included the discovered quisviruses and one representative per ICTV-recognized virus family of the orders *Sobelivirales* (3 virus families), *Picornavirales* (9), and *Nidovirales* (14) from the class *Pisoniviricetes,* a divergent unclassified picorna-like virus, as well as genus representatives of the orders *Patatavirales* (3 virus genera from the family *Potyviridae*) and *Stellavirales* (2 from the family *Astroviridae*) from the class *Stelpaviricetes* into the phylogenetic analysis (Supplementary Table S2). We constructed a multiple sequence alignment of the RdRp amino acid sequences demarcated by motifs G and E (Gorbalenya et al. 2002, Peersen 2017) at, respectively, the N- and C-terminus within the Viralis software (Gorbalenya et al. 2010). From this alignment, we extracted all columns (*n* = 208) that contained <10% gaps for phylogenetic analysis. The best-fitting evolutionary model was selected using ModelTest-NG version 0.2.0 (Darriba et al. 2020), which was the BLOSUM62 + I + G4 model. A maximum likelihood phylogenetic tree of the RdRp proteins was reconstructed using PhyML version 20120412 with parameters ‘-d aa -m BLOSUM62 -f m -v e -a e -c 4 -o tlr’ (Guindon and Gascuel 2003). Branching support was assessed using the Shimodaira–Hasegawa (SH)–like procedure (Shimodaira and Hasegawa 1999).

### Helicase similarity network

Multiple sequence alignments of helicase superfamilies SF1–6 were obtained from either Pfam/InterPro (SF6 RuvB: accession PF05496), CDD (SF4 Gp4D: accession CD19483, SF5 Rho: accession CD01128), PROSITE (SF3 of DNA viruses: accession PS51206) (Hulo et al. 2006, Marchler-Bauer et al. 2017, Mistry et al. 2021), or in-house from Viralis (SF1, SF2, SF3 of RNA viruses). For SF2, we included helicases from *Potyviridae* and *Flaviviridae* and flavi-like viruses. For SF3, we generated two alignments encompassing proteins from (i) *Caliciviridae* and calici-like viruses and (ii) *Picornaviridae* and picorna-like viruses. Scores obtained from the comparison of the two SF3 multiple sequence alignments serve as a positive control.

The multiple sequence alignments for SF1–6 and the quisvirus ATPase alignment were subjected to pairwise HMM-vs-HMM comparisons using hhalign in (default) local mode with parameter ‘-M first’ from the HHblits package (Remmert et al. 2012). For each pair of alignments, we considered two comparisons treating an alignment either as query or as template in the hhalign analysis. From these two comparisons, we collected the Sum_probs values, which integrate over the posterior probabilities of all aligned pairs of match states. To obtain a final score for the alignment pair, we took the maximum of these two Sum_probs values (maximum sum of poster probabilities, MSPP). The MSPP values were scaled by multiplying them with a factor of 0.025 for all alignment pairs (Supplementary Table S3) and used as edge weights in a similarity network of the helicases, which was visualized in R using the igraph package (Antonov et al. 2026).

### Protein 3D structure modelling

Protein tertiary structures were predicted using AlphaFold3 Server (Abramson et al. 2024). We conducted two independent modelling runs for each input sequence and considered the five top-ranking structures. In total, 10 structures were aligned and visualized using ChimeraX to assess prediction variability (Goddard et al. 2018). We aligned the (putative) ATPase structures of *Strandella quadrimaculata* quisvirus (accession IAMJ01023800) and representatives for SF3 (2C of poliovirus 1; NP_041277), SF4 (Gp4D of Prochlorococcus phage P-RSP2; AGF91554), SF5 (transcription termination factor Rho *Chlorobaculum tepidum* TLS; NP_661168), and SF6 (Holliday junction branch migration DNA helicase RuvB of *Bdellovibrio bacteriovorus*; WP_011164893) using Kpax with the -multi option (Ritchie 2016). This structural alignment was used by Kpax to generate a multiple sequence alignment for these five ATPases, which was manually refined around globally conserved residues. The multiple sequence alignment was visualized using Jalview (Clamp et al. 2004).

### Sequence analysis of reads for testing a model of discontinuous subgenomic RNA synthesis and reconstructing putative transcription regulatory sequences

To test a model of discontinuous transcription, we combined conventional computational tools in an original procedure including statistical evaluation of read distribution along the virus genome. We expected the presence of chimeric reads that partially align to two distantly located genomic regions for the discontinuous but not the continuous transcription model. These regions include the leader TRS (L-TRS) and body TRS (B-TRS) in nidoviruses.

Specifically, chimeric reads were initially detected during manual quality check of reads aligning to the assembled viral genome sequences. We performed blastn searches (default parameters) of both genomic termini and regions spanning the first AUG triplet of each potential ORF of 99 aa or more, downstream of ORF1ab. Reads that mapped upstream of any qualifying 3′-terminal ORF were collected and checked to determine if a part of the read mapped to the genomic 5′-untranslated region upstream of ORF1a (leader). Read counts were made by collecting all reads matching the genomic 5′-end leader region, mapping back to the genome using blastn, and tallying reads composed of three portions: with at least 15 nt of sequences unique for the 5′-genomic leader and 3′-body, in addition to any putative crossover region common to both leader and body.

We then expanded our procedure to analyse all reads available for a given viral genome sequence in the respective SRA dataset in an automated manner within a probabilistic framework. We screened the SRA datasets for the presence of chimeric reads that partially align upstream of the ORF1a start codon and to a 181-nt region around a 3′ORF start codon, respectively, in the reconstructed genome sequence. Such reads would be expected if a subgenomic RNA is leader-containing and 5′-coterminal with the genomic RNA. As a negative control, we applied the same approach (outlined in the following) to each AUG triplet in three reading frames of the genomic sequence in 5′ to 3′ direction. Sequencing reads were mapped to a window of [−120, +60] nt around an AUG triplet using Burrow-Wheeler Aligner (BWA)-MEM Version: 0.7.17-r1188 (Li and Durbin 2009). The aligned reads were extracted from the resulting BAM file using SAMtools Version 1.18 (Danecek et al. 2021). These latter reads were re-aligned to the full viral genome sequence excluding the original AUG window. The reads that re-aligned upstream of the ORF1a start codon were determined (based on the information on their alignment position in the BAM file) and counted, resulting in read counts RC_k_ for each AUG window k. The total count of these reads, RC_total_, was calculated as the sum over all RC_k_ values. For each AUG window, a binomial test was applied to calculate a *P*-value in R using the function binom.test(x = RC_k_, n = RC_total_, p = w/L, alternative = "greater") with w being AUG window size (181 nt) and L being genome size minus length of the 5′UTR. A *P*-value smaller than 0.05 was considered to be statistically significant. The results were visualized in R using the ggplot2 library (Wickham 2016).

To verify the developed procedure, we applied it to positive and negative controls run along with new datasets (see Results). We used SARS-CoV-2 Wuhan-Hu-1 (Genbank accession NC_045512.2) and the original sequencing dataset (SRR10971381) (Wu et al. 2020) as a positive control with extensively studied discontinuous transcription (Snijder et al. 2016, Kim et al. 2020). As a negative control, we used a nidovirus from the family *Roniviridae*, Gill-associated virus (GAV), whose genome sequence was *de novo* assembled from the sequencing run SRR6868128. Unlike vertebrate nidoviruses, GAV has been shown to produce all subgenomic RNAs leader-less through continuous transcription (Cowley et al. 2002).

Finally, we proceeded to identify junctions of two genomic loci and putative TRSs in subgenomic RNAs that could be produced by discontinuous transcription. We extracted the parts of the chimeric reads that map to both genomic loci in the reference genome sequence (immediately upstream of ORF1a and upstream of each 3′ORF), based on their CIGAR (Compact Idiosyncratic Gapped Alignment Report) strings. These read subsequences for all 3′ORFs were then aligned using MAFFT version 7.505 with default parameters (Katoh and Standley 2013). Sequence logos were created using WebLogo (Crooks et al. 2004).

## Results

### Discovery of a new deeply rooted lineage in the phylum *Pisuviricota*

From a Data-Driven Virus Discovery (DDVD) (Lauber and Seitz 2022, Lauber et al. 2024, Neuman et al. 2025) screen for RNA viruses in 30 105 arthropod transcriptome sequencing projects from the SRA, we initially identified two novel and closely related viral genomes. They diverged profoundly from all known RNA viruses and showed only remote RdRp sequence similarity to members of the order *Picornavirales*. The two viral genomes were found in spider sequencing projects of *Nephilengys cruentata* and *Pardosa pseudoannulata* (Supplementary Table S1). We used the predicted RdRp-encoding proteins of these two viral genomes as query in a BLAST search against the TSA database and identified four additional and unannotated viral genomes of this virus group in data from the 1000 spider silkomes (Arakawa et al. 2022) projects of *S. quadrimaculata, Clubiona zilla, Octonoba sybotides*, and *Haplodrassus kanenoi*. We only obtained hits against spider projects and no hits in other arthropods.

### Genome architecture and protein annotation of new viruses

Based on the presence of terminal noncoding sequences, 5′-UTR and 3′-UTR, and other features (see below), we considered four of the six novel viral genomes to be coding-complete with estimated genome sizes ranging from 20 474 to 22 035 nt, while two are partial (12 005, 14 810 nt) (Supplementary Table S1). The complete genomes have a multi-ORF organization including at least five ORFs flanked by 5′- and 3′-UTRs. The two largest and overlapping ORFs, ORF1a, and ORF1b, account for ~80% of the genome size. We identified −1 programmed ribosomal frameshifting (-1 PRF) elements with predicted slippery sequence AAAAAAC or UUUAAAC in the conserved ORF1a/b overlap of all six viruses (Supplementary Fig. S1). We predicted two alternative and comparably supported pseudoknot RNA structures downstream of the shift site at, respectively, ~5 or 13 nt (Supplementary Fig. S1), indicating that the -1 PRF site predictions may require further research. ORF1b is followed by four to six partly overlapping predicted ORFs of sizes in the range of 101–661 codons (Supplementary Table S4). Translation of ORF1a and ORF1b from the genomic RNA may produce two polyproteins, termed pp1a (from ORF1a) and pp1ab (ORF1a and ORF1b), like in nidoviruses.

We used amino acid multiple sequence alignments of the six viruses to scan different sequence and structural databases in profile mode for genome annotation (see Methods). Based on significant hits (E-value < 10^−3^), we identified six putative nonstructural protein domains that are conserved across the six viruses (Supplementary Table S5). They include cysteine papain-like protease (PLpro) and cysteine 3CLpro in pp1a, as well as four domains downstream of −1PRF in the pp1ab. They are putative RdRp, RNA genome expansion and replicase-associated (REXA) domain (Neuman et al. 2025), novel AAA+ ATPase, and DNA/RNA demethylase AlkB-like domain (Moore and Meng 2019) (Supplementary Figs S2 and S3). A potential trans-membrane (TM) domain has been identified upstream of quisvirus 3CLpro (Supplementary Table S5); these two domains are linked also in the 2B–3Cpro module of picornaviruses. Somewhat similar, top hits for five enzymes PLpro, 3CLpro, RdRp, REXA, and AlkB were predominantly observed against ssRNA+ virus proteins, mostly from the phylum *Pisuviricota*, and specifically family *Picornaviridae*. Hit aa conservation varied greatly, from very high and domain-wide (up to E-value 10^−13^ and 90% coverage, AlkB) to moderate (RdRp and REXA) to limited around the active centre (PLpro and 3CLpro) (Supplementary Table S5). In all five enzymes, known essential residues are conserved, indicating that they are functional. We describe results for the novel AAA+ ATPase in a separate section below.

Based on characterization of other viruses, we propose that pp1a/pp1ab are autoproteolytically processed to mature proteins by 3CLpro with possible assistance of PLpro. The cleavage sites in six viruses may be conserved but remain unknown. Accordingly, the exact boundaries/sizes and identity of mature proteins of these viruses are yet to be established.

**Figure 1:**
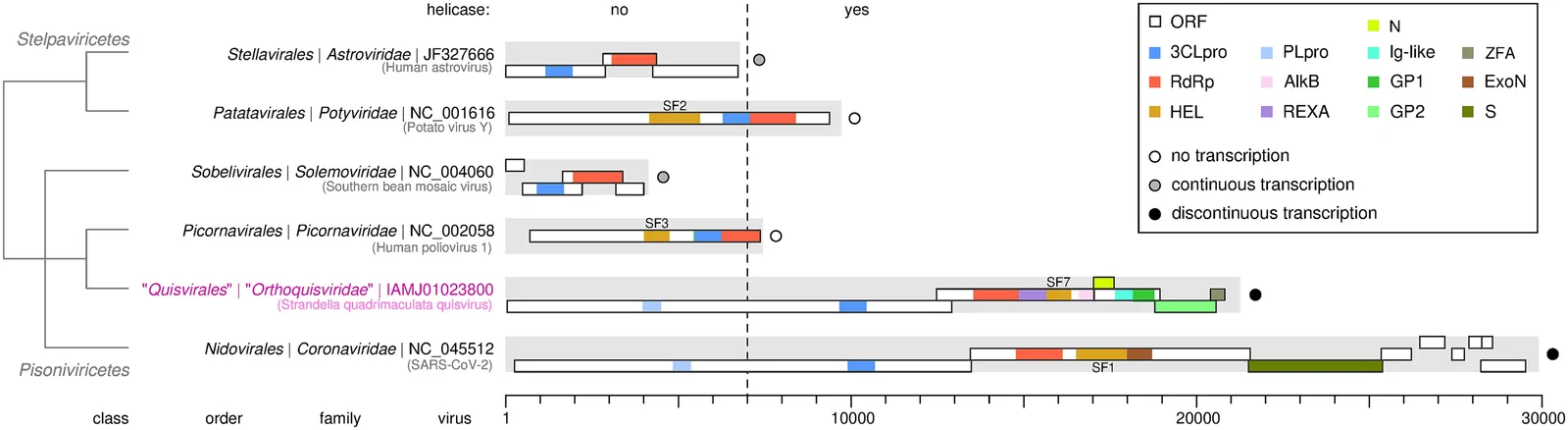
Quisvirus and selected *Pisuviricota* representatives. Shown are genome maps for a quisvirus (purple font) and one representative for each recognized virus order in the phylum *Pisuviricota* whose members encode a 3C-like protease (blue) and an RdRp (red) domain. Some of the viruses also encode a helicase/ATPase domain (yellow). For the *S. quadrimaculata* quisvirus representing ‘*Quisvirales*’, ORFs of at least 150 nt in length are shown and predicted domains are coloured: nucleocapsid protein (N), glycoprotein (GP) domains GP1 and GP2, immunoglobulin-like domain (Ig-like), and zinc finger–associated domain (ZFA). Genome and ORF sizes are drawn to scale; the dashed vertical line indicates a genome size of 7 kb, separating viruses with and without a helicase. Borders of protein domains are estimates based on either annotation in Genbank records or hits of profile HMM-based sequence homology searches. Utilization and type of genome transcription is indicated through circles to the right of a genome map (see inlet legend); note that members of the family *Caliciviridae* from the order *Picornavirales* employ continuous transcription. The cladogram at the left is based on the classification of the included orders in the ICTV taxonomy and the phylogenetic analysis in this study (Fig. 2); branch lengths have no meaning.

**Figure 2:**
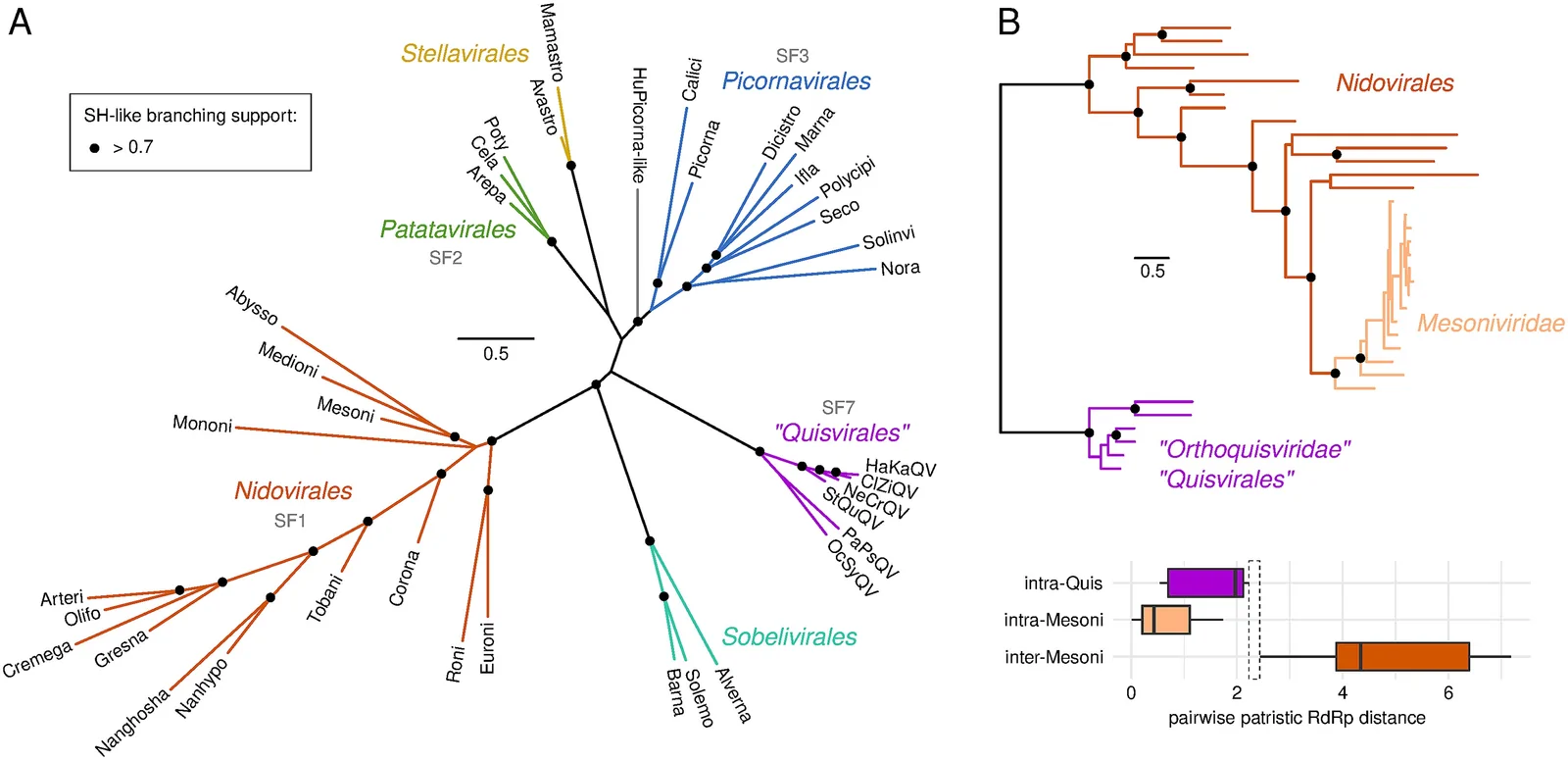
Phylogenetic relationships of quisviruses with other members of the phylum *Pisuviricota* encoding the 3CLpro-RdRp domain pair. (A) The maximum likelihood RdRp-based phylogeny was reconstructed using PhyML. Quisviruses, as well as ICTV-recognized orders *Picornavirales, Nidovirales, Sobelivirales, Patatavirales,* and *Stellavirales,* as well as an unclassified virus (grey)*,* are discriminated by coloured branches. One representative virus per ICTV-recognized virus family or genus was included in the analysis (Supplementary Table S2). Dots at internal branching events indicate SH-like support values larger 0.7; otherwise, no dot is shown. The scale bar is in average number of amino acid substitutions per site. The included quisviruses are: *C. zilla* quisvirus (ClZiQV; accession: AWI01044675), *H. kanenoi* quisvirus (HaKaQV; IBAC01001151), *N. cruentata* quisvirus (NeCrQV; SRR3943478), *O. sybotides* quisvirus (OcSyQV; IAZZ01033660), *P. pseudoannulata* quisvirus (PaPsQV; SRR22498918), *S. quadrimaculata* quisvirus (StQuQV; IAMJ01023800). For ICTV virus orders, one representative per reference virus family or genus is shown: *Sobelivirales*: *Solemoviridae* (Solemo; Southern bean mosaic virus; NC_004060.2), *Barnaviridae* (Barna; Mushroom bacilliform virus; NC_001633.1), *Alvernaviridae* (Alverna; Heterocapsa circularisquama RNA virus; NC_007518.1); *Picornavirales*: *Noraviridae* (Nora; Nora virus; NC_007919.3), *Polycipiviridae* (Polycipi; Lasius neglectus virus; NC_035450.1), *Secoviridae* (Seco; Camellia virus A; NC_077103.1), *Solinviviridae* (Solinvi; Nylanderia fulva virus 1; NC_030651.1), *Picornaviridae* (Picorna; Poliovirus; NC_002058.3), *Dicistroviridae* (Dicistro; Cricket paralysis virus; NC_003924.1), *Iflaviridae* (Ifla; Deformed wing virus; NC_004830.2), *Marnaviridae* (Marna; Heterosigma akashiwo RNA virus; NC_005281.1), *Caliciviridae* (Calici; Norwalk virus; NC_001959.2); *Nidovirales*: *Tobaniviridae* (Tobani; Breda virus; NC_007447.1), *Coronaviridae* (Corona; Severe acute respiratory syndrome coronavirus 2; NC_045512.2), *Abyssoviridae* (Abysso; *Aplysia californica* nido-like virus; NC_040711.1), *Mesoniviridae* (Mesoni; Nam Dinh virus; NC_015874.1), *Medioniviridae* (Medioni; Botrylloides leachii nidovirus; NC_055538.1), *Mononiviridae* (Mononi; Planarian secretory cell nidovirus; NC_040361.1), *Euroniviridae* (Euroni; Beihai hermit crab virus 4; NC_032490.1); *Roniviridae* (Roni; Yellow head virus; NC_043505.1), *Gresnaviridae* (Gresna; Guangdong greater green snake gresnavirus; NC_046959.1), *Cremegaviridae* (Cremega; *Trionyx sinensis* hemorrhagic syndrome cremegavirus; NC_076637.1), *Arteriviridae* (Arteri; lactate dehydrogenase–elevating virus; NC_001639.1), *Olifoviridae* (Olifo; Hainan *Oligodon formosanus* olifovirus; NC_046958.1), *Nanghoshaviridae* (Nanghosha; Nanhai ghost shark nanghoshavirus; NC_046960.1); *Nanhypoviridae* (Nanhypo; Wuhan japanese halfbeak nanhypovirus; NC_046957.1); *Potyvirus* (Poty; Potato virus Y; U09509); *Celavirus* (Cela; Celery latent virus; MH932227), *Arepavirus* (Arepa; Areca palm necrotic spindle spot virus; MH330686); *Mamastrovirus* (Mamastro; Human astrovirus type 1; Z25771); *Avastrovirus* (Avastro; Avastrovirus 1; Y15936); unclassified (HuPicorna-like; Hubei picorna-like virus 69; NC_033021). Helicase superfamilies (SF) are indicated per virus order. (B) RdRp subtree for ‘*Quisvirales*’ and *Nidovirales* members from (A) and additionally including all available RefSeq sequences for the family *Mesoniviridae* (top). This tree was used to extract patristic distances for each pair of quisviruses (intra-Quis), each pair of mesoniviruses (intra-Mesoni), and each pair of a mesonivirus and a nidovirus from another family (inter-Mesoni); the distribution of these pairwise distances are shown as boxplots with a region devoid of any pairwise distances highlighted by a dashed rectangle (bottom).

In addition to the nonstructural domains, we identified two putative structural domains of presumed virus particles in the ORFs downstream of ORF1b that are conserved in all six quisviruses. This includes two glycoprotein (GP)-like domains in, respectively, the C-terminal part of ORF3 (GP1) and in ORF4 (GP2) (Supplementary Table S5). Both domains include a putative TM region at the C-terminus, and 8 and 10 conserved Cys residues, respectively; these features are common for GPs of enveloped RNA virus particles (Li 2016, Hulswit et al. 2021) (Supplementary Fig. S4). Moreover, an immunoglobulin-like (Ig-like) domain was found upstream of GP1 in ORF3 of four quisviruses which are relatively closely related to each other but not in *O. sybotides* quisvirus and *P. pseudoannulata* quisvirus that form two basal lineages in the RdRp phylogeny (Supplementary Fig. S4, Fig. 2). All six quisviruses share a poorly conserved domain of unknown function upstream of the Ig-like domain. It is encoded in either ORF3 or an alternative reading frame of a short ORF2 (Fig. 1). Provisionally, we designated ORF2 as the nucleocapsid locus (N) due to characteristic amino acid composition of the encoded protein, although we did not observe significant sequence similarity to reference database entries. At the extreme 3′-terminus, another small ORF5 of under 300 nt in length slightly overlaps ORF4. It encodes a protein with a conserved 60 aa core region flanked by highly variable terminal sequences. The core subdomain has four conserved Cys and His residues indicative of a zinc finger, which we provisionally named zinc finger–associated (ZFA) domain. These results suggest that quisviruses employ enveloped virions, like nidoviruses and unlike picornaviruses, in line with the plausible nidovirus-like sgRNA expression of the involved ORFs (see below).

### A new order ‘*Quisvirales*’ in the class *Pisoniviricetes*

The observed 3CLpro-RdRp domain segregation was previously described in three orders of the class *Pisoniviricetes* and two orders of the class *Stelpaviricetes* (all in the phylum *Pisuviricota*) (Fig. 1). When analysed in the RdRp-based phylogeny of the class *Pisoniviricetes*, which included a prototype virus from each recognized virus family in the class, the six novel viruses formed a divergent, monophyletic lineage. This lineage was positioned marginally closer to the order *Picornavirales,* compared to the other two orders, *Sobelivirales* and *Nidovirales*, although further analysis may be required to resolve the deep branching patterns (Fig. 2A).

Based on the RdRp phylogeny, we propose that these viruses form a new RNA virus order in the class *Pisoniviricetes*. Due to a surprising combination of several familiar and highly unusual features of the novel viruses, we provisionally named this novel order ‘*Quisvirales*’, referring to the Latin word ‘quis’, which translates to ‘What is this?’. The pairwise evolutionary distances in the RdRp between the six quisviruses is at a scale observed for the family *Mesoniviridae* (order *Nidovirales*) that have viruses with a similar range of genome sizes (20.0–22.0 kb) (Fig. 2B). We therefore propose that the described quisviruses form a provisional virus family that we named ‘*Orthoquisviridae*’. The six quisviruses may be prototypes of six different virus species according to their considerable genetic divergence (Fig. 2B).

### Quisviruses may encode a novel type of ATPases that resembles single AAA+/RecA-like helicases

**Figure 3:**
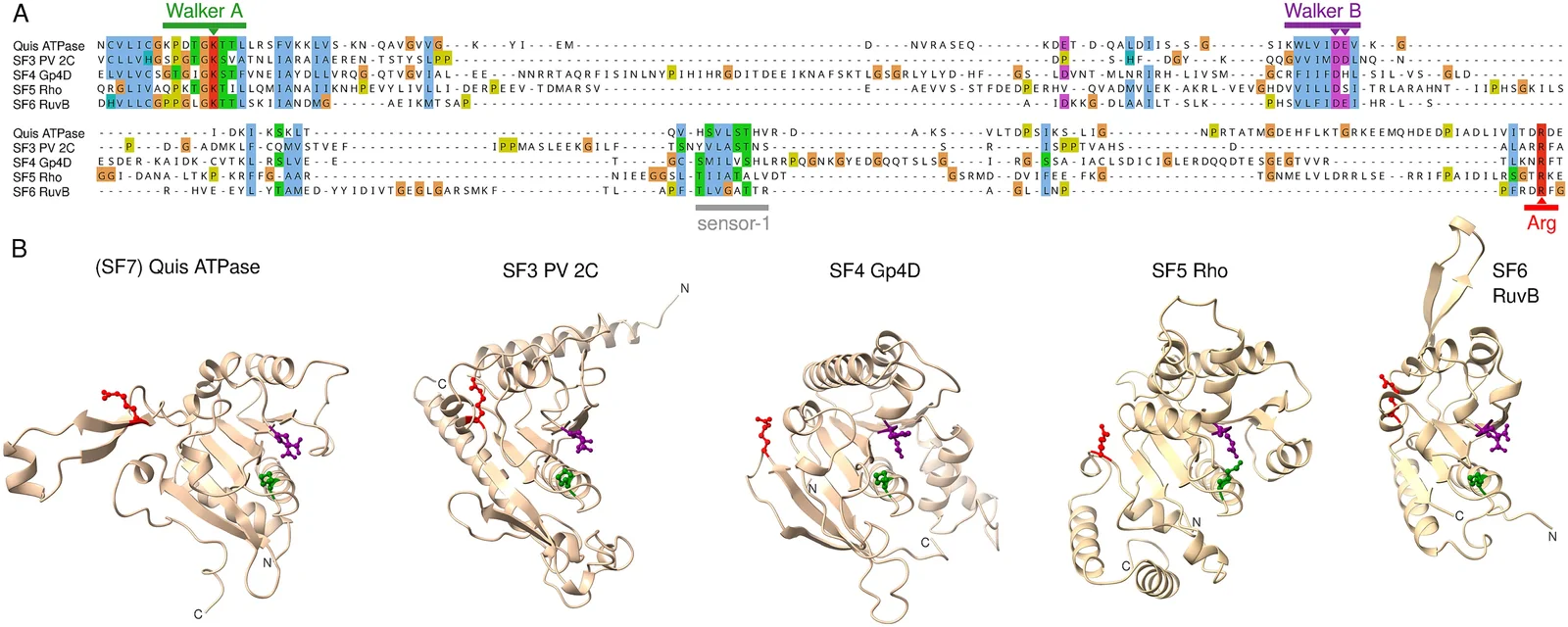
Sequence and structural features of the quisvirus ATPase are indicative of RecA-like structure and helicase activity. (A) Multiple sequence alignment of quisvirus ATPase (IAMJ01023800) and superfamily 3 2C helicase of poliovirus 1 (SF3 PV 2C; Genbank accession NP_041277), superfamily 4 Gp4D helicase of Prochlorococcus phage P-RSP2 (SF4 Gp4D; AGF91554), superfamily 5 transcription termination factor Rho of *C. tepidum* TLS (SF5 Rho; NP_661168), and superfamily 6 Holliday junction branch migration DNA helicase RuvB of *B. bacteriovorus* (SF6 RuvB; WP_011164893). The sequence alignment was generated using Kpax and is based on a superposition of predicted tertiary structures of the proteins. The N- and C-terminal parts of the alignment are not shown for simplicity. The alignment was visualized using Jalview with Clustal colouring mode. (B) The predicted structures of quisvirus ATPase and SF3–6 helicases were superimposed and are shown side-by-side. Four conserved residues in three of four motifs indicated by rectangles in panel (A) are highlighted by colour; green: K in Walker A; purple: DE in Walker B; red: R in arginine finger; grey: Sensor-1.

Based on a strong link between the presence of a cognate helicase in ssRNA+ viruses with large genome size (>7.9 kb) and the similarities in genomic organization to nidoviruses, we expected to identify an SF1 helicase domain in pp1ab of quisviruses in our sequence-vs-profile searches. Surprisingly, we observed no hits against SF1–SF2 profiles and only moderately supported hits against SF3–SF4 profiles that were limited to Walker box A and B and that are common for a wide range of ATPases (Supplementary Table S6). This analysis identified a new ATPase (~190aa) downstream of and close to the RdRp, the position that is occupied by a much larger SF1 helicase (HEL1) in nidoviruses (Figs 1 and 3).

We annotated this ATPase based on hits to 114 Pfam entries and many hundreds of entries in other databases with hit E-values from 10^−3^ to 10^−11^ (Supplementary Table S7). While the majority of hits were limited to the Walker A NTPase motif, there were significant hits against AAA+ domains in several bacterial uncharacterized multi-domain proteins that encompassed >75% of query and target domains (UniRef100, E-value from 10^−9^ to 10^−11^; the longest hit is 173aa to A0A022Q4Y0, 2.1 × 10^−10^). AAA+ domains facilitate many functions, including helicases of SF3–6 and those unclassified with single AAA+/RecA-like fold. Accordingly, the longest hits for this ATPase were of 170 aa with SF4 DNA B helicases (PF03796, E-value 3.1 × 10^−7^) and 163 aa with SF4 helicase domains of *E. coli* phage T7 DNA primase/helicase (pdb70 entry 1CR0_A, E-value 2.1 × 10^−6^) and Twinkle (Riccio et al. 2022) mtDNA replisome (8E2L_B, E-value 1.1 × 10^−4^) (Supplementary Table S7). Hit alignments between the query and targets (templates) included four SF4 motifs of the template (Fig. 3A).

Also, the quisvirus ATPase includes conserved sequence motifs (Walker A, Walker B, sensor-1, arginine finger) that are involved in ATP binding and hydrolysis, as well as energy coupling to unwinding in known helicases (Fig. 3A). Upon structural comparison of SF3–SF6 helicases and a quisvirus ATPase tertiary structural model (Supplementary Fig. S5A), key residues of these sequence motifs occupied spatially highly similar positions in a single AAA+/RecA-like domain, implying functional similarity (Fig. 3B). Accordingly, the quisvirus ATPase exhibits highest (although moderate) sequence affinity to members of the ring-forming single-AAA+/RecA-like SF3-SF4 in a network analysis (Supplementary Table S3) in which the weights of connecting edges are proportional to MSPP scores obtained in pairwise profile HMM-based sequence comparisons (Supplementary Fig. S5B). Namely, the MSPP score for quisvirus ATPase was in the range of 25.6–85.6 (mean MSSP of 46.9) against other SFs, comparable to that between DNA- and RNA-virus SF3 domains (67.4–70.3) and also to inter-SF MSPP scores (Supplementary Table S3). Notably, the MSPP scores involving the quisvirus ATPase were considerably lower than that of the positive control (126.6) of comparing SF3 domains of calici-like and picorna-like viruses (Supplementary Fig. S5B). Combined, these results suggest that the quisvirus ATPase is the prototype of a new helicase superfamily (SF7) and has helicase activity.

### Discontinuous genome transcription may produce subgenomic RNAs in quisviruses

We observed a sharp and step-wise increase of read coverage depth at the 3′ genome region for five of the six viruses with the highest mean coverage (Supplementary Fig. S6). This sequencing coverage depth pattern is highly similar to that seen for nidoviruses (Lauber et al. 2024, Neuman et al. 2025) and other multi-ORF ssRNA+ viruses with differential transcription control of genome expression (Goddard et al. 2018). It suggests that the newly discovered viruses express the ORFs downstream of ORF1b in the 3′ genome region (3′ORFs) *via* sg RNAs.

**Figure 4:**
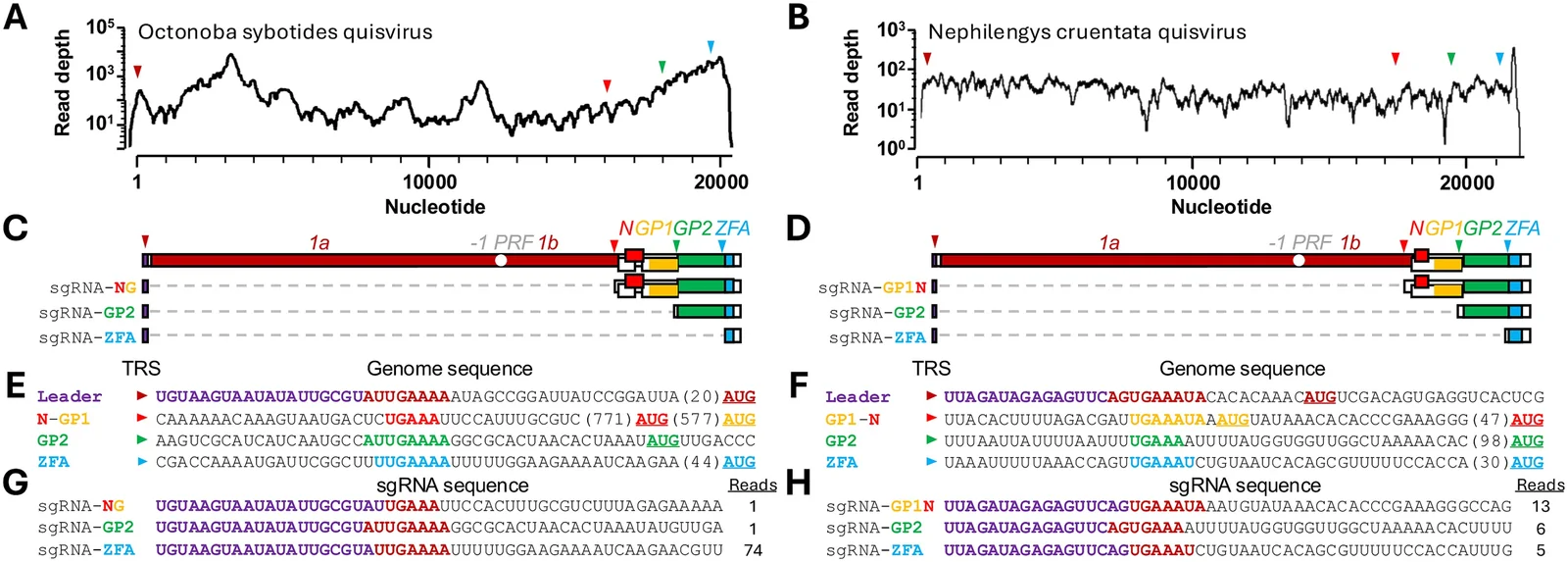
Evidence for subgenomic RNAs produced through discontinuous transcription in two quisviruses. Sequence read depth maps (A, B), schematics showing sgRNAs in relation to genes (C, D), putative transcription-regulatory sequences (TRS; E, F) and sequences containing subgenomic RNA leader-body junctions (G, H) are shown for *Octonoba sybotides* quisvirus (A, C, E, G) and *Nephilengys cruentata* quisvirus (B, D, F, H). Inferred start codons are underlined and colour-coded to match the genome maps with predicted protein domains highlighted: nucleocapsid protein (N), glycoproteins GP1 and GP2, and zinc finger–associated (ZFA) domain. Detailed depictions of genomic organization are shown in Supplementary Fig. S6 for six quisviruses.

Given the parallel between quisvirus and nidoviruses (see above), we proceeded to test whether the sg RNAs may also be 5′-coterminal with the genomic RNA and produced by discontinuous transcription, as in nidoviruses. To this end, we screened the SRA datasets for the presence of chimeric reads that partially align upstream of the ORF1a start codon and to a 180-nt region (‘window’) around each of the 3′ORF start codons, respectively, using an original procedure (see Methods). We reasoned that these matches of chimeric reads would identify leader and body of transcripts if no comparable hits were observed for AUG triplets elsewhere in the genome. Indeed, we observed such chimeric reads for all or all except one 3′ORFs of the four quisviruses with nearly complete genome sequences. They led us to infer junctions of two separate genomic loci that invariably included the leader-containing 5′-ends of their subgenomic RNAs (Fig. 4**,**  Supplementary Fig. S7). Such chimeric reads were preferentially observed for genomic regions in close proximity to 3′ORF start codons compared to other AUG triplets across the viral genome and this difference received significant statistical support (Supplementary Figs S8 and S9). When sequences of all these AUG-containing regions were compared for each virus, we identified a recurrent oligonucleotide sequence in vicinity of the junctions, (U)UGAAA(U/A), that resembled the core sequence of coronavirus TRSs, indicating a similar role for the quisviruses (Fig. 4, Supplementary Figs S7 and S10). Importantly, our developed procedure correctly identified chimeric reads for most leader-containing subgenomic RNAs encoding the 3′-terminal ORFs of SARS-CoV-2 serving as a positive control, and it did not produce any comparable signals for a ronivirus (GAV) that employs continuous transcription and thus was used as a negative control (Supplementary Fig. S11). Combined, these results strongly suggest that the quisvirus subgenomic RNAs coding for the 3′ORFs are generated through a discontinuous transcription mechanism that is guided by TRSs.

## Discussion

In this study, we introduce a provisional order ‘*Quisvirales*’ comprising six RNA viruses discovered in the metatranscriptomic ‘dark matter’ of spiders. Like nidoviruses, quisviruses may employ discontinuous transcription. Also, they encode replicase polyproteins of nidovirus-like complexity and deviate from all known ssRNA+ viruses with comparably large genome sizes in respect to the omnipresent helicase. Quisviruses either don’t encode a helicase domain or encode a helicase different from known SF1–SF6 helicases of all life forms (favoured by our analysis). Both the linkage between helicase presence and RNA-genome size, and the helicase-superfamily classification were proposed several decades ago (Gorbalenya and Koonin 1989, Gorbalenya and Koonin 1993), and, to date, these relationships have been consistently reinforced despite the rapid pace of genomic and helicase characterization in numerous cellular organisms and viruses. Thus, and regardless of which of two hypotheses proves correct, the discovered quisviruses already revise the established relationship between RNA viruses and helicases.

We acknowledge that further biochemical and structural characterization of the quisvirus proteome is essential to resolve the residual uncertainty. However, demonstration of helicase activity may take years of work as researchers have learned with SF3 helicases of picornaviruses, most relevant to this study while remaining provisional (Sweeney et al. 2010, Shankar et al. 2024). This ongoing experience may be due to technical reasons since SF3 helicase activity was observed for DNA viruses (Hickman and Dyda 2005). Also, it is in contrast to the successful demonstration of ATPase activity and ring structure (Pfister and Wimmer 1999, Papageorgiou et al. 2010, Guan et al. 2017), two other genomic-based inferences that, however, are shared with other enzymes (Gorbalenya and Koonin 1993, Singleton et al. 2007). Due to these considerations, we did not pursue experimental characterization of helicase activity in this study. Instead, we discuss below how the presented findings already advance our understanding of ssRNA+ viruses and why quisviruses encoding a new SF helicase seems most plausible.

To start with, we reiterate that our results are reliable, consistent with literature, original, and informative, even if technically they are limited to various bioinformatics analyses. We applied a DDVD approach (Lauber and Seitz 2022) to initiate this study and observed statistical support for two assembled virus genomes in respect to RNA read depth and coverage breadth as derived from (infected) spider transcriptomes (Supplementary Fig. S2). These assembly metrics are comparable to those reported in our other high-throughput virus genomic discoveries (Lauber et al. 2024, Neuman et al. 2025). Furthermore, four other closely related genome sequences were identified in the TSA and reassembled. The six new viruses share a multi-ORF genomic organization and encode several protein domains with characteristic sequence motifs typical for ssRNA+ viruses.

Due to the deep separation of this new group of viruses from the most closely related viruses in a RdRp-based tree and unique but conserved features of genome architecture, we propose a new tentative order provisionally named ‘*Quisvirales*’, class *Pisoniviricetes*, phylum *Pisuviricota*. ‘*Quisvirales*’ and *Nidovirales* seem to be most similar, since four other orders of the 3CLpro-RdRp encoding viruses (*Picornavirales, Sobelivirales, Stellavirales, Patatavirales*) have much smaller genomes and use naked capsids (Krishna 2005, Zell et al. 2017, Sõmera et al. 2021, Inoue-Nagata et al. 2022). The 20–22 kb genome sizes of quisviruses are exceeded only by flavi-like viruses and nidoviruses, while they resemble those of the nidovirus family *Mesoniviridae* (Nga et al. 2011, Zirkel et al. 2011, Zirkel et al. 2013, Shi et al. 2016b). The observed quisvirus genome size range is narrow for a virus order and may reflect a currently limited sampling, virus family-wise (‘*Orthoquisviridae*’) and host-wise (Araneae), rather than the natural genome size range of the entire order ‘*Quisvirales*’. For comparison, genome sizes of the multi-family *Picornavirales* and *Nidovirales* vary from two- to almost five-fold. It is particularly interesting to find out whether quisviruses with even larger genome sizes exist, given that these viruses don’t share a proof-reading exoribonuclease (ExoN) with the 19–64 kb nidoviruses (Smith et al. 2014, Neuman et al. 2025). Moreover, an increased sampling may verify or expand the current host range of quisviruses that is limited to different species of spiders. Conspicuously, metatranscriptomes of a much larger spectrum of invertebrates were found quisvirus-negative while nidovirus-positive (Neuman et al. 2025). This observation indicates that the observed virus-host link for quisviruses and Araneae may be biologically sound.

Strikingly, our analysis of chimeric reads in four datasets from which nearly complete viral genomes were assembled indicate that quisviruses generate subgenomic RNAs that are 5′-coterminal with the genomic RNA and thus leader-containing. This resembles subgenomic RNAs produced by several nidovirus families and contrasts with those of other transcription-dependent ssRNA+ viruses (Miller and Koev 2000, Sztuba-Solińska et al. 2011, Sola et al. 2015), strongly suggesting that quisviruses utilize discontinuous transcription for subgenomic RNA production. In line with this conclusion and with two exceptions, we identified leader and body TRSs that are conserved across the viruses described here. The apparent lack of leaders for two subgenomic RNAs in two viruses may be attributed to low read coverage depth of these viral genomes. Alternatively, these subgenomic RNAs may be synthesized by continuous transcription, like demonstrated for a torovirus that uses continuous and discontinuous transcription for different subgenomic RNAs (Van Vliet et al. 2002). Yet another possibility is that two overlapping ORFs in few quisviruses may be expressed from a single subgenomic RNA by diverse mechanisms of translational control (Firth and Brierley 2012). Due to feasibility issues, we did not analyse reads for complementary (negative) RNAs, which are produced in small molar quantities while serving as templates for the respective subgenomic RNAs in nidoviruses (Sawicki et al. 2007). Together, these findings unveil quisviruses as being only the second virus order after the *Nidovirales* that utilize discontinuous transcription. Comparative analysis of viruses from these two orders may provide unique insights into benefits and mechanisms of discontinuous transcription.

Interestingly, quisviruses and a provisional family-like group of spider-infecting nidoviruses uniquely share a pair of adjacent REXA and AlkB domains that are encoded in proximity to the RdRp in the viral genome (Supplementary Table S5) (Neuman et al. 2025). Both domains may affect RNA replication and/or transcription. AlkB may contribute to genomic repair by demethylation of oxidized nucleotides (Van Den Born et al. 2008). REXA remains an uncharacterized putative ATPase that is genetically segregated in variable locations with the RdRp of many invertebrate nidoviruses and flavi-like viruses (Neuman et al. 2025). In contrast, quisviruses have the largest ssRNA+ genomes that don’t encode a methyltransferase, commonly recruited for cap methylation of the 5′-terminal nucleotide of virus RNAs (Decroly et al. 2012, Mushegian 2022). It is therefore conceivable that quisviruses possess a protein covalently linked to the 5′-terminus of their RNAs, enabling protein-primed RNA synthesis by the RdRp, which is typical for the phylum (Paul and Wimmer 2015, Eruera et al. 2021).

Also, REXA and AlkB flank the novel AAA+ ATPase domain in pp1ab of quisviruses, and this segregation may be functionally important. In nidoviruses, this AAA+ ATPase locus is occupied by a SF1 helicase. We did not observe top hits to helicases of SF1–SF6 for quisvirus ATPase or another domain, indicating that quisviruses differ from other ssRNA+ viruses with genomes larger than 7.9 kb. Like homo-oligomeric ring helicases of SF3–SF6, the quisvirus ATPase appears to adopt a single AAA+/RecA-like fold and shares four key sequence motifs with these helicases (Fig. 3). In the pairwise sequence distance space, this ATPase has diverged from the canonical helicase superfamilies as much as they have from each other (Supplementary Fig. S6B). Collectively, our results are consistent with the quisvirus ATPase being a helicase, possibly of a new superfamily SF7 comparable to SF3–SF6.

Only SF1 and SF2 helicases, composed of two paralogous RecA domains, are involved in virtually every genome replication and expression process of all cellular organisms and are found in DNA as well as in RNA viruses (Gorbalenya and Koonin 1993, Fairman-Williams et al. 2010). In contrast, the utilization of the homo-oligomeric SF3–SF6 is much more restricted among biological entities and in different processes (Hickman and Dyda 2005, O’Shea and Berger 2014, Peter and Falkenberg 2020). The novel SF7 fits the SF3-SF6 profile and that may help rationalize its restricted host range and late discovery. Since SF1 and SF2 include many families recognized on different grounds, SF3–SF7 could be placed as separate families in a single SF, emulating the original depiction of their relationship (Gorbalenya and Koonin 1993). It was later modified to the currently used helicase classification purely for the sake of nomenclature uniformity (Singleton et al. 2007).

The ‘*Quisvirales*’ is only the second RNA virus order after the *Picornavirales* that encodes a single-AAA+/RecA-like helicase, and its discovery revises the known upper size of the respective virus RNA genomes (Supplementary Table S8). Strikingly, this putative quisvirus helicase and SF3 helicases of the *Picornavirales* differ mostly in sizes of sequence spacers separating four key motifs rather than in the motifs. In contrast to the *Picornavirales* counterpart, the putative quisvirus helicase is encoded in the unusual genomic position downstream of the RdRp (as in nidoviruses), which may have affected its role in genome replication, while its expression may be attenuated by the upstream −1 PRF. The available results are compatible with a singular acquisition of a single-AAA+/RecA-like helicase by an ancestral 3CLpro-RdRp encoding virus either upstream or downstream of the RdRp. During subsequent macroevolution, this helicase might have been relocated to the other genomic locus in virus progeny, and the ancestral and descendant helicases gave rise to SF3 and SF7 of the two ssRNA+ virus orders *Picornavirales* and ‘*Quisvirales*’. Other evolutionary scenarios, including independent acquisitions of helicases in ancestors of the two orders, may be envisioned as well. We acknowledge that some of the moderate hits in our profile-based searches of the quisvirus ATPase to uncharacterized ORFs may suggest a more widespread distribution of SF7 helicases and did not rule out a possible origin of the ancestral enzyme in bacteria or other microorganisms.

While quisviruses encoding a novel SF7 helicase seems the most plausible scenario, these viruses might use the AAA+/RecA-like domain for a function other than nucleic acid unwinding during replication. However, this alternative scenario would place quisviruses apart from all other known ssRNA+ viruses, making quisviruses even more unconventional than presented under the helicase model. Regardless of the actual function of the novel ATPase, the discovery of quisviruses offers unexpected insights into the linkage of RNA virus genome size and helicases and informs studies of the orders *Picornavirales* and *Nidovirales*. The genomic architecture and proteome of quisviruses implies that large replicase polyproteins of nidovirus-like complexity evolved repeatedly from an ancestral 3CLpro–RdRp-encoding RNA virus lineage in the phylum *Pisuviricota*. Moreover, both viral lineages appear capable of supporting discontinuous transcription, representing a notable revision of the current paradigm.

## Supplementary Material

Supplementary_material_veag052

## Data Availability

Transcriptomic data used in this study are publicly available through the SRA and TSA databases at the NCBI. Data accompanying the study, including computational models of protein tertiary structures, have been uploaded to FigShare: DOI 10.6084/m9.figshare.31119181. Viral genome sequences assembled in this study will also be made available through the NCBI Genbank database upon publication. The Virushunter and Virusgatherer tools are available on GitHub: https://github.com/lauberlab/VirusHunterGatherer.
