# Supplementary material for: Discovery of the order ‘Quisvirales’ redefines the evolution of RNA replication and transcription in the phylum Pisuviricota
Source: Virus Evol. 2026 Aug 18;12(1):veag052. doi: 10.1093/ve/veag052 (PMC13548321; doi:10.1093/ve/veag052)
Supplement: Supplementary file 1 [file supplementary_material_veag052.zip › FigureS6.pdf]

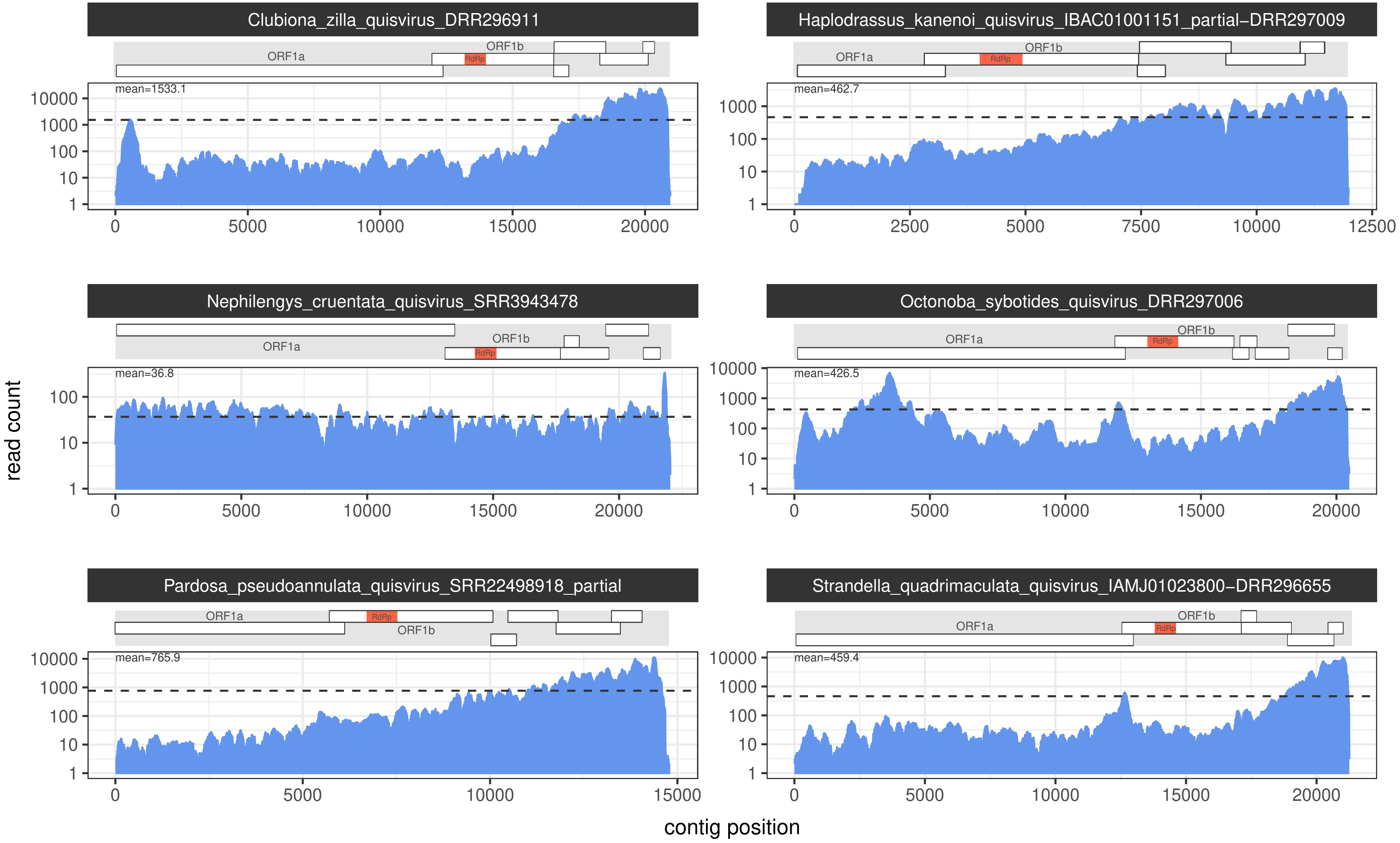

Supplementary Figure 6. Sequencing read coverage depth of six quisvirus genome assemblies. Genome maps with open reading frames (ORFs, white rectangles) are shown above the coverage plots. ORFs 1a and 1b and position of the RdRp domain (red) is indicated.
