# Supplementary material for: Discovery of the order ‘Quisvirales’ redefines the evolution of RNA replication and transcription in the phylum Pisuviricota
Source: Virus Evol. 2026 Aug 18;12(1):veag052. doi: 10.1093/ve/veag052 (PMC13548321; doi:10.1093/ve/veag052)
Supplement: Supplementary file 1 [file supplementary_material_veag052.zip › FigureS7.pdf]

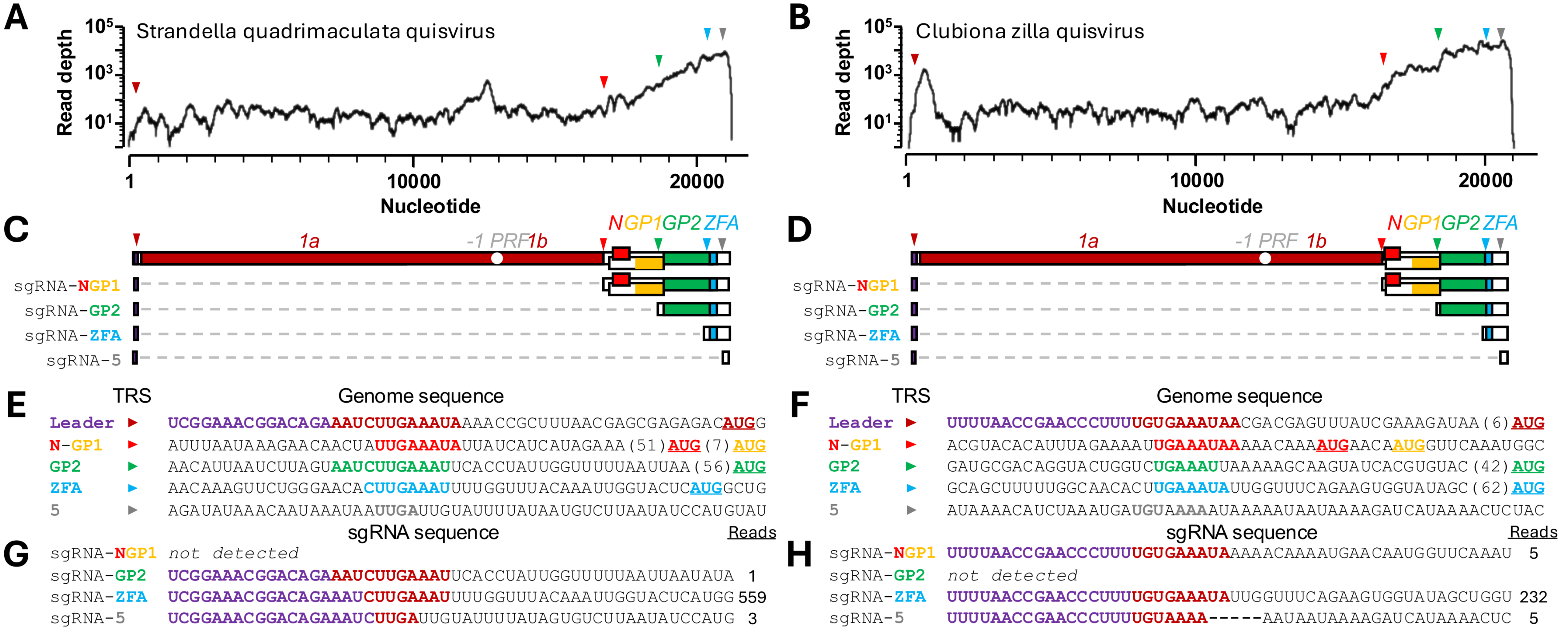

Supplementary Figure 7. Additional evidence for quisvirus subgenomic RNA production. Coloring and labeling conventions are as described in the legend for main Figure 4. Non-coding sgRNA 5 and its inferred TRS are marked in grey.
