# Supplementary material for: Discovery of the order ‘Quisvirales’ redefines the evolution of RNA replication and transcription in the phylum Pisuviricota
Source: Virus Evol. 2026 Aug 18;12(1):veag052. doi: 10.1093/ve/veag052 (PMC13548321; doi:10.1093/ve/veag052)
Supplement: Supplementary file 1 [file supplementary_material_veag052.zip › FigureS8.pdf]

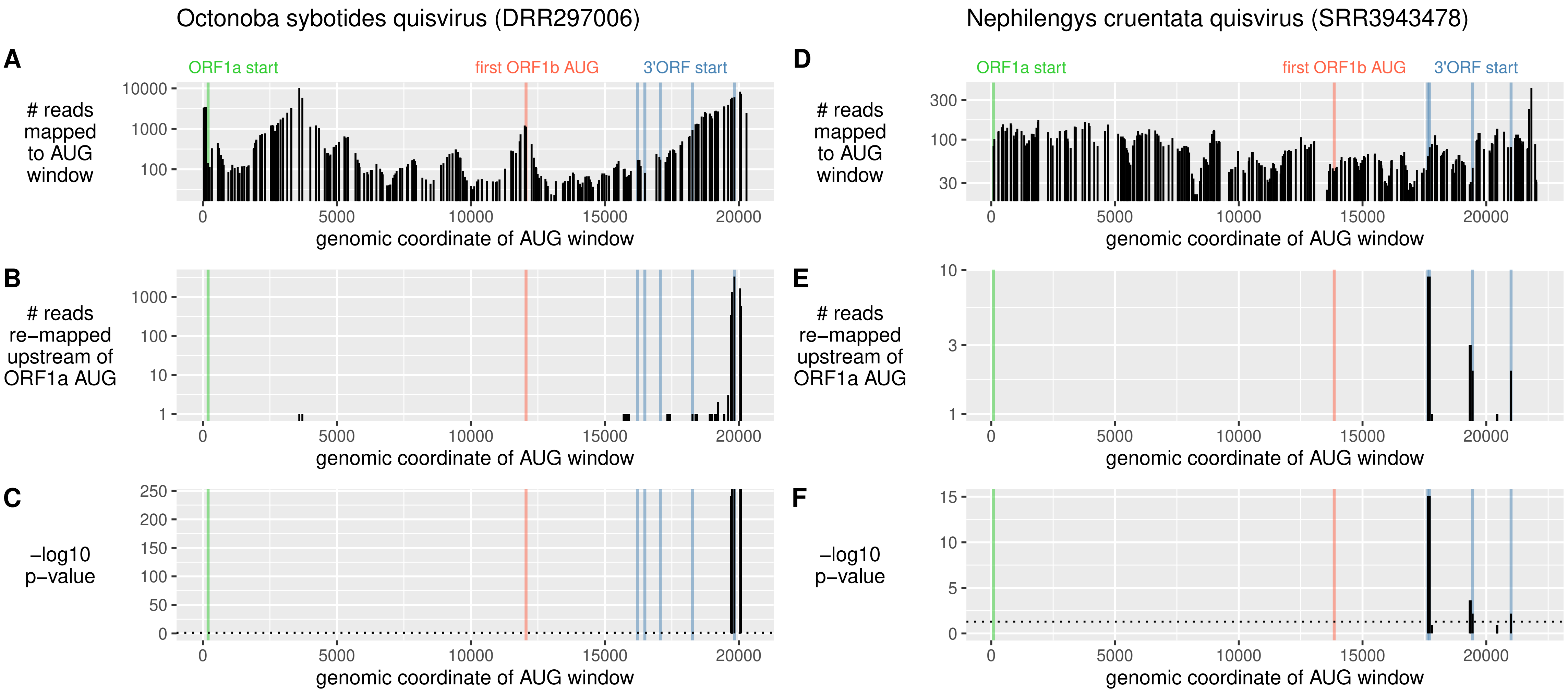

Supplementary Figure 8. Analysis of chimeric reads that may result from leader-containing subgenomic RNAs produced by discontinuous transcription for Octonoba sybotides quisvirus (left column) and Nephilengys cruentata quisvirus (right column). Accessions of used SRA datasets are shown in brackets. Sequencing reads were mapped to a window of [-120,+60] nt around each AUG triplet in three reading frames of the genome (plus polarity), and the observed read count per window was plotted against its position in the genome sequence (A, D panels). This included both reads aligning along their entire length and soft-clipped reads (partially mapped with terminal overhang). For each AUG window, the aligning reads were collected for a second analysis in which they were re-mapped to the other parts of the genome by excluding the original AUG window from consideration. The reads that re-mapped upstream of the ORF1a start codon were identified as “chimeric”, counted for each AUG window separately, and plotted against the position of their AUG window in the genome sequence (B, E). Statistical significance of the obtained numbers were evaluated by p-values using a binomial test (see Methods for details); the dotted line corresponds to a p-value of 0.05 (C, F). In all plots, the start codon or the first AUG codon of an ORF are highlighted by color: ORF1a (green), ORF1b (red), other ORFs downstream of ORF1b (blue).
