# Supplementary material for: Discovery of the order ‘Quisvirales’ redefines the evolution of RNA replication and transcription in the phylum Pisuviricota
Source: Virus Evol. 2026 Aug 18;12(1):veag052. doi: 10.1093/ve/veag052 (PMC13548321; doi:10.1093/ve/veag052)
Supplement: Supplementary file 1 [file supplementary_material_veag052.zip › FigureS1.pdf]

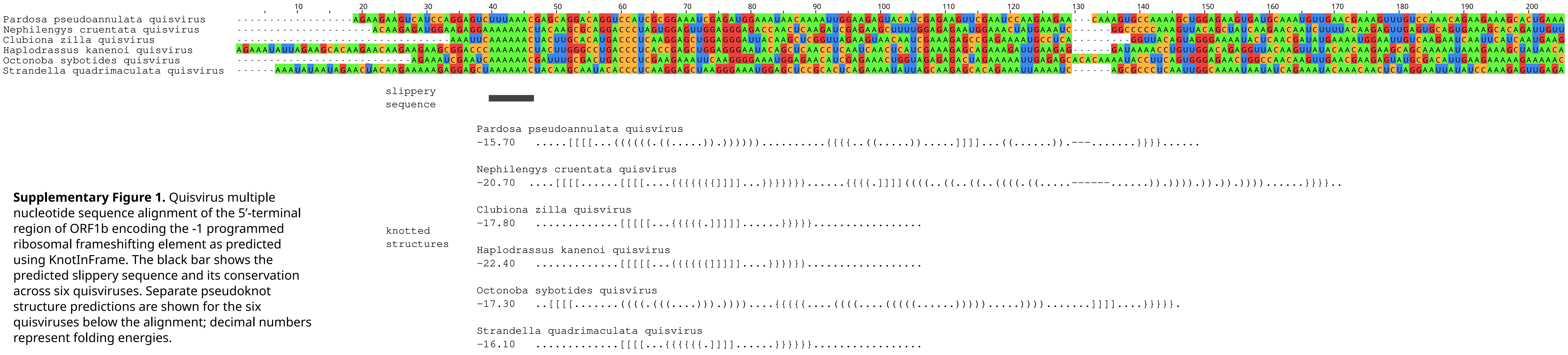

Supplementary Figure 1. Quisvirus multiple nucleotide sequence alignment of the 5′-terminal region of ORF1b encoding the -1 programmed ribosomal frameshifting element as predicted using KnotInFrame. The black bar shows the predicted slippery sequence and its conservation across six quisviruses. Separate pseudoknot structure predictions are shown for the six quisviruses below the alignment; decimal numbers represent folding energies.
