# Supplementary material for: Discovery of the order ‘Quisvirales’ redefines the evolution of RNA replication and transcription in the phylum Pisuviricota
Source: Virus Evol. 2026 Aug 18;12(1):veag052. doi: 10.1093/ve/veag052 (PMC13548321; doi:10.1093/ve/veag052)
Supplement: Supplementary file 1 [file supplementary_material_veag052.zip › FigureS2.pdf]

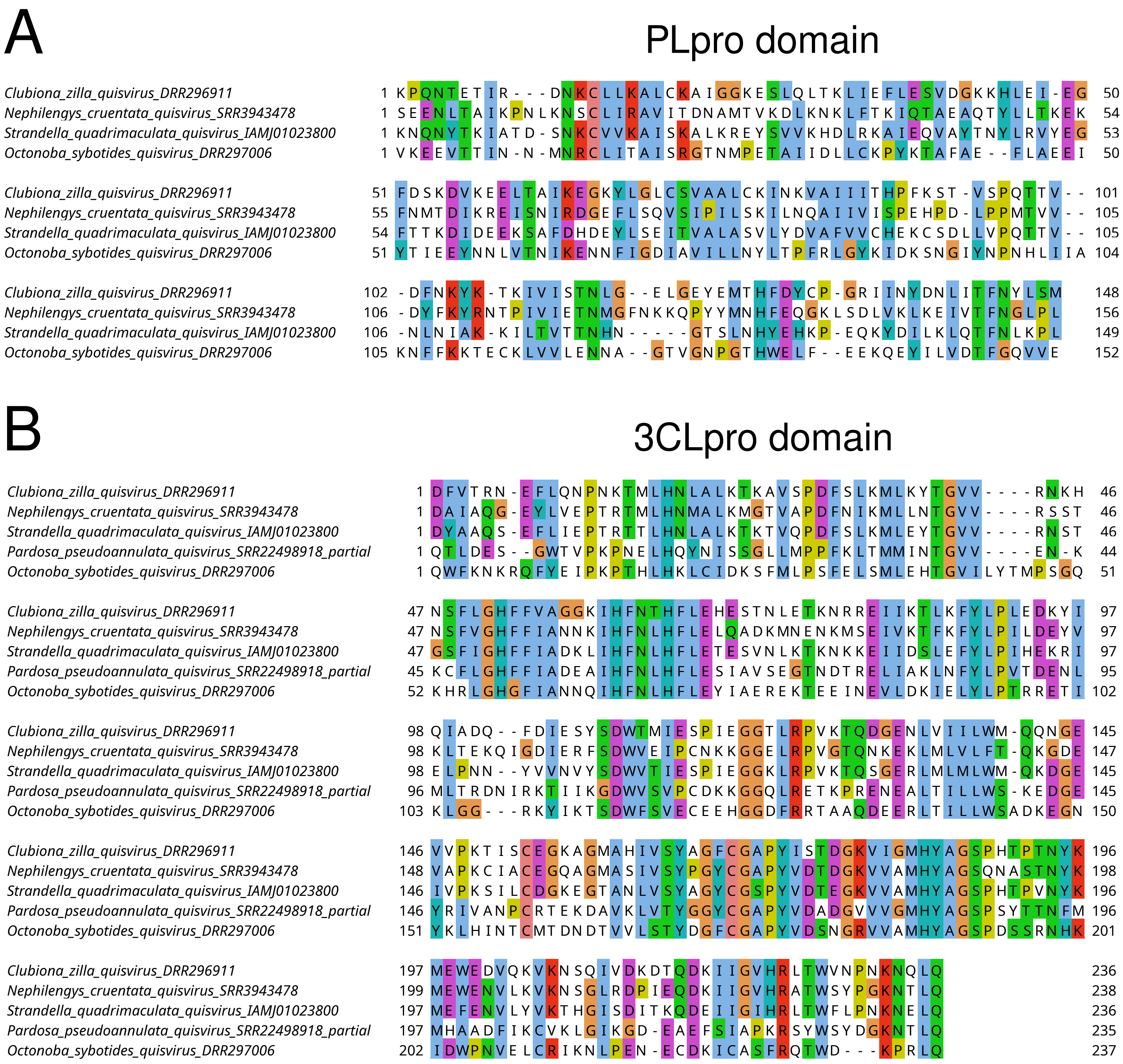

Supplementary Figure 2. Quisvirus multiple amino acid sequence alignments of PLpro (A) and 3CLpro (B) domains.
