# Supplementary material for: Discovery of the order ‘Quisvirales’ redefines the evolution of RNA replication and transcription in the phylum Pisuviricota
Source: Virus Evol. 2026 Aug 18;12(1):veag052. doi: 10.1093/ve/veag052 (PMC13548321; doi:10.1093/ve/veag052)
Supplement: Supplementary file 1 [file supplementary_material_veag052.zip › FigureS3.pdf]

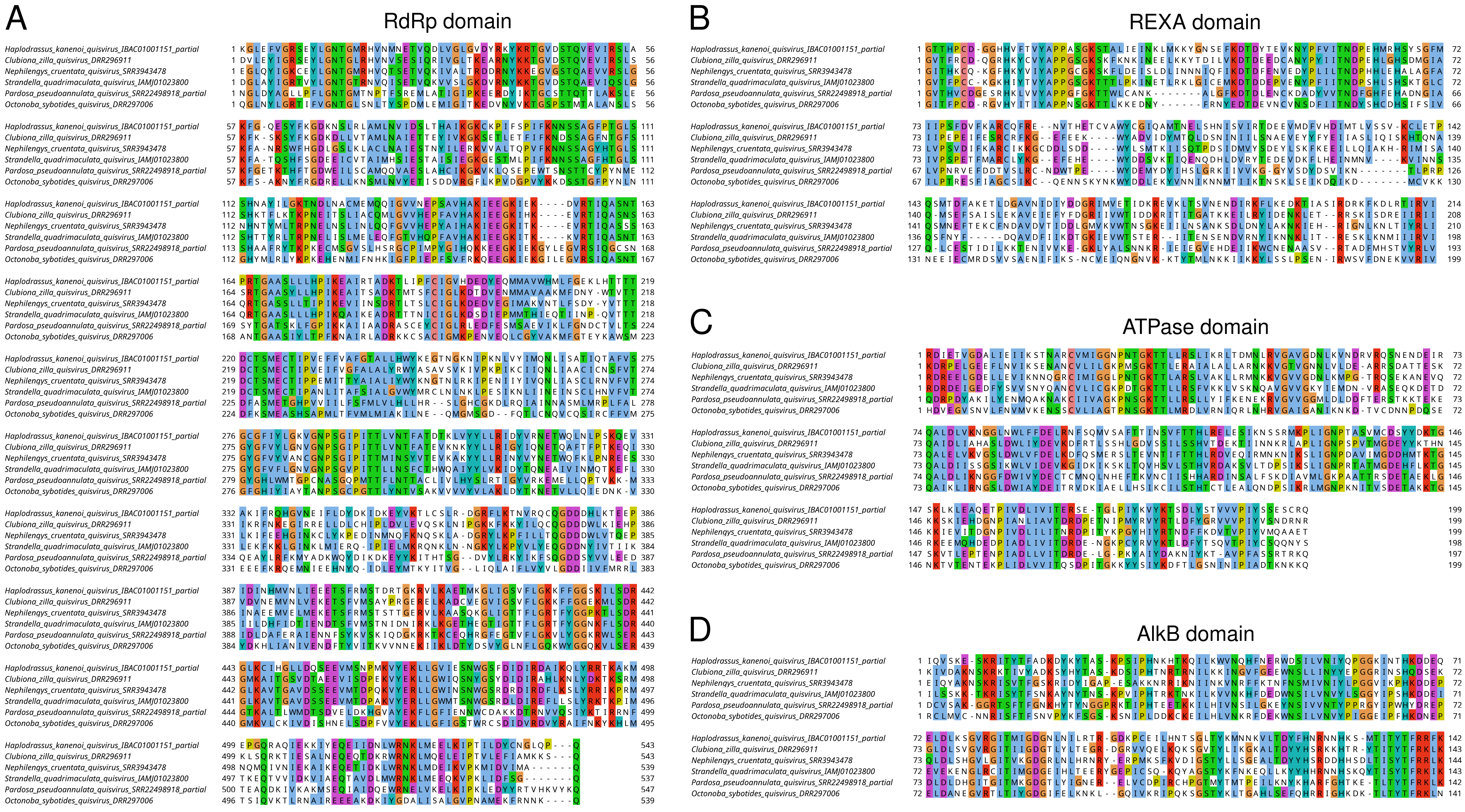

Supplementary Figure 3. Quisvirus multiple amino acid sequence alignments of RdRp (A), REXA (B), SF7 ATPase (C) and AlkB (D) domains.
