# Supplementary material for: Discovery of the order ‘Quisvirales’ redefines the evolution of RNA replication and transcription in the phylum Pisuviricota
Source: Virus Evol. 2026 Aug 18;12(1):veag052. doi: 10.1093/ve/veag052 (PMC13548321; doi:10.1093/ve/veag052)
Supplement: Supplementary file 1 [file supplementary_material_veag052.zip › FigureS5.pdf]

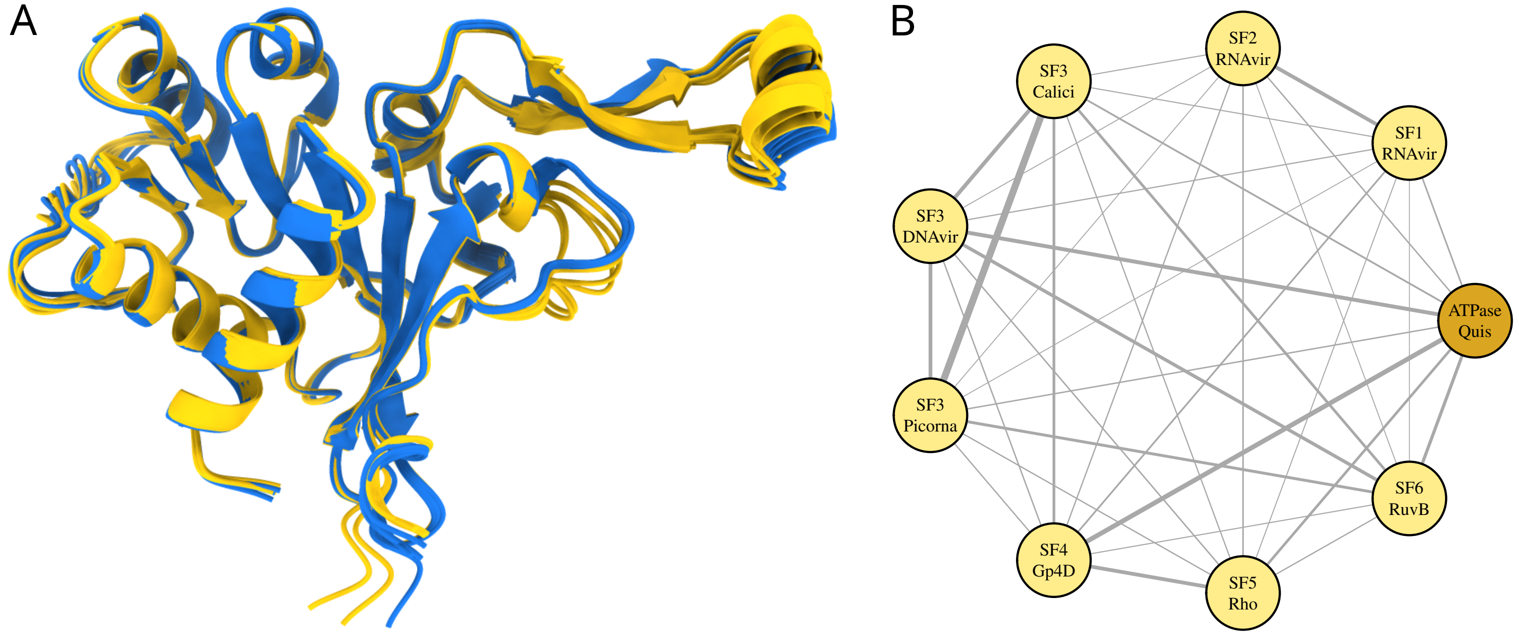

Supplementary Figure 5. (A) Superimposition of 10 top-ranking quisvirus SF7 helicase models from two independent AlphaFold3 runs; blue: five models from first run, yellow: five models from second run. (B) Network of helicase superfamilies. Nodes represent the quisvirus SF7 helicase (ATPase) or representatives of helicase superfamilies SF1-SF6 o RNA viruses (RNAvir), *Caliciviridae* and calici-like viruses (Calici), *Picornaviridae* and picorna-like viruses (Picorna), DNA viruses (DNAvir) or cellular organisms (see Methods for further details). The widths of the connecting edges are proportional to the maximum sum of posterior probabilities (MSPP) of hits obtained in pairwise HMM-vs-HMM comparisons using HHalign (see Methods for details and Table S3). The comparison of "SF3 Calici" vs. "SF3 Picorna" serves as a positive control.
